# Exploring the pathogenesis of canine epilepsy using a systems genetics method and implications for anti-epilepsy drug discovery

**DOI:** 10.18632/oncotarget.23719

**Published:** 2017-12-27

**Authors:** Ze-Jia Cui, Ye-Mao Liu, Qiang Zhu, Jingbo Xia, Hong-Yu Zhang

**Affiliations:** ^1^ Hubei Key Laboratory of Agricultural Bioinformatics, College of Informatics, Huazhong Agricultural University, Hubei, Wuhan, China

**Keywords:** canine, systems genetics, pathogenic factors, drug combinations

## Abstract

Epilepsy is a common neurological disorder in domestic dogs. However, its complex mechanism involves multiple genetic and environmental factors that make it challenging to identify the real pathogenic factors contributing to epilepsy, particularly for idiopathic epilepsy. Conventional genome-wide association studies (GWASs) can detect various genes associated with epilepsy, although they primarily detect the effects of single-site mutations in epilepsy while ignoring their interactions. In this study, we used a systems genetics method combining both GWAS and gene interactions and obtained 26 significantly mutated subnetworks. Among these subnetworks, seven genes were reported to be involved in neurological disorders. Combined with gene ontology enrichment analysis, we focused on 4 subnetworks that included traditional GWAS-neglected genes. Moreover, we performed a drug enrichment analysis for each subnetwork and identified significantly enriched candidate anti-epilepsy drugs using a hypergeometric test. We discovered 22 potential drug combinations that induced possible synergistic effects for epilepsy treatment, and one of these drug combinations has been confirmed in the Drug Combination database (DCDB) to have beneficial anti-epileptic effects. The method proposed in this study provides deep insight into the pathogenesis of canine epilepsy and implications for anti-epilepsy drug discovery.

## INTRODUCTION

Epilepsy is a common serious neurological disease, which does latent harm to human health [1]. Treatments of epilepsy are currently limited to drugs that provide only symptomatic control of seizures. Current treatments have adverse effects and are ineffective in up to 40% of patients [2]. However, the pathogenesis of epilepsy, and the resistance to available antiepileptic drugs remains poorly understood [1]. Though a better understanding of the genetic targets and mechanisms of anti-seizure drug activity offers trusty direction in drug selection for individual patients, the experiments in need are ethically and technically difficult. For this reason, animal models are useful in anti-seizure drug research [3]. One such model organism is the dog, because both humans and companion animals suffer from epilepsy, which is a chronic neurologic disorder affecting nearly 50 million people and 0.5–5% of the canine population worldwide [4]. Conversely, models of anti-seizure medication targets and activities that are derived from humans are useful in epilepsy research in a canine context.

Domestic dogs share many parallel syndromes with humans [4]. Evolutionarily speaking, similarities in medical genetics between dogs and humans can be found throughout history. Dogs evolved from the gray wolf in East Asia at least 15,000 years ago, co-operationally integrated with mankind, and developed a mutually beneficial relationship over a long period of time. Many dog breeds have shown a high prevalence of specific diseases, such as blindness, cancer, heart disease, and epilepsy [5], most of which are also common in humans. Furthermore, the clinical manifestations of the two species are often similar [6]. High-quality draft genome sequencing, has verified a degree of similarity of 96% between dog and human genes [7]. Therefore, the domestic dog is treated as a valuable model species for human diseases [4], and canine epilepsy pathogenesis is regarded as the same as that of human epilepsy. Thus, human anti-epilepsy drugs are promising agents for canine therapy.

Even prior to the problems of selection of anti-seizure medications, diagnosis of epilepsy is complicated. Generally, at least two unprovoked seizures, or one seizure combined with additional symptoms, e.g., adverse head injury, previous stroke or an abnormality on an electroencephalogram (EEG) brain scan, are required for diagnosis [8]. However, it is more difficult than that in some cases, the cause of most cases of epilepsy is unknown [9]. Generally speaking, canine epilepsy can be divided into reactive seizures, structural epilepsy, and idiopathic epilepsy according to etiology [10]. The mechanism of reactive seizures is the response of the brain to abnormal systemic metabolism, such as hypoglycemia, hypocalcemia, uremia, poisoning and so on. Structural epilepsy is mainly due to abnormal brain structure, including brain abnormalities caused by hydrocephalus, craniocerebral tumor causing compression of brain parenchyma, trauma caused by brain injury or damage, infectious diseases such as toxoplasmosis, rabies, tetanus and other pathogens into the brain tissue, or destruction of the normal structure of brain tissue. Idiopathic epilepsy or primarily genetic epilepsy, which is associated with congenital or genetic factors, is the most common neurological disorder in dogs [11]. However, the mechanism of idiopathic epilepsy remains unclear.

Anti-epileptic drugs (AEDs) have long been administered to humans and dogs. Early in 1857, bromide was introduced to treat epilepsy [12]. It was combined with potassium or sodium to form a crystal powder. Currently, there are over 20 anti-epileptic drugs on the market for treating humans. These include carbamazepine (also known as carbatrol, equetro, tegretol), gabapentin (neurontin), levetiracetam (keppra), lamotrigine (lamictal), oxcarbazepine (trileptal), pregabalin (lyrica), tiagabine (gabitril), topiramate (topamax), and valproate (depakote, depakene). The first three types of drugs are also widely used in dogs. Furthermore, there are also AEDs for dogs, such as clorazepate, diazepam, felbamate, phenobarbital, phenytoin, primidone, valproic acid, and zonisamide (http://www.canine-epilepsy.com/AEDs.html). Though many new drugs have been developed that work well in humans, their efficacies decrease in canine treatment because dogs metabolize these drugs much more quickly, making new drug discovery for dogs challenging.

The recent availability of a large number of canine genetic samples presents an opportunity to realize the promise of using understanding of the genetic targets and mechanisms of anti-seizure drug activity to help in drug selection for individual patients. A promising approach to finding these genetic things is the Genome-Wide Association Study (GWAS) [13]. The first GWAS on focal epilepsy was carried out with 3,445 patients and 6,935 controls from Northern Europe. No significant genetic markers were found, as none of the single nucleotide polymorphisms (SNPs) corresponded to *P*-values that reached significance after Bonferroni correction [14]. Flank expansion of 95 and 116 kb from the top SNP only led to a predicted pseudogene. A second GWAS study on 1,000 patients from China identified two highly correlated variants in CAMSAP1L1 (CAMSAP1-Like 1), a calmodulin-regulated spectrin-associated protein expressed in neurons and astrocytes in the mammalian nervous system [15]. Although each GWAS study thus far has offered a list of candidate genes, few of these genes have been functionally validated. For most patients, epilepsy is regarded as a complex disorder associated with multiple genes and external environmental factors, which also makes it difficult to rely on single-gene associations. Therefore, it is hoped that earlier findings can be enhanced by additional GWAS results and next-generation DNA sequencing (NGS) [16].

Prior to the GWAS, 10 genes had been reported as underlying canine epilepsy [4]. Among these, 9 genes are associated with progressive myoclonic epilepsy in several dog breeds, i.e., *EPM2B* [17], *CLN8* [18], *CLN5* [19], *CTSD* [20], *TPP1* [21], *PPT1* [22], *ARSG* [23], *CLN6* [24], and *ATP13A2* [25], and one gene, *LGI2*, is associated with genetic epilepsy [26]. In 2015, using GWAS, Hayward *et al.* [27] collected the largest set of canine GWAS results. Among these results, the authors found additional candidate genes related to epilepsy in Irish Wolfhounds using 34 cases and 168 controls. However, very little is currently known about the genetic factors that are involved here. Several research efforts are currently focused on the discovery of key genes associated with epilepsy, although the results remain far from unveiling the entire mechanism. This concern leads to our current research.

Among published idiopathic epilepsy studies that have investigated inheritance patterns, various breeds show evidence of autosomal recessive inheritance. Many of these studies also demonstrated polygenic inheritance, indicating that the genetic basis of idiopathic epilepsy is complex in dogs [4]. In this paper, we used a hybrid method combining GWAS and HotNet2 with Hayward *et al.*’s data [27] and found 26 significant subnetworks correlated with canine epilepsy. Combined with gene ontology (GO) enrichment analysis, our method not only identified *PPFIA1*, *ASB5*, *SLC1A2* and *MCU* to correspond to significant *P*-values in the GWAS results but also identified the pathogenic genes *PTPRM*, *PTPRD* and *SGCE*, which were omitted by the GWAS analysis. Among the above seven genes, *PPFIA1* [28], *SLC1A2* [29], *PTPRM* [30] and *SGCE* [31] were reported to be associated with epilepsy, and the others were associated with mental illness [32–34]. Moreover, drug enrichment analysis showed that the subnetworks we identified are capable of enriching anti-epilepsy drugs. In view of the combination therapy in cancer enhances efficacy and reduces drug resistance compared to the mono-therapy approach [35], we believe that this strategy can work on epilepsy. When targets of different anti-epilepsy drugs are enriched in the same subnetwork, their combinations are also more likely to effectively treat epilepsy. Ultimately, 22 drug combinations were obtained, one of which was confirmed in the Drug Combination database (DCDB, https://omictools.com/drug-combination-database-tool). The results of this study showed the effectiveness of our method in exploring the pathogenesis of canine epilepsy, and this approach increases cost efficiency for anti-epilepsy drug discovery.

## RESULTS AND DISCUSSION

### Replication of the GWAS experiment

MLogit [36] was used to analyze the GWAS data. Our experiment completely replicated the results of the previous GWAS and achieved high consistency.

**Figure 1:**
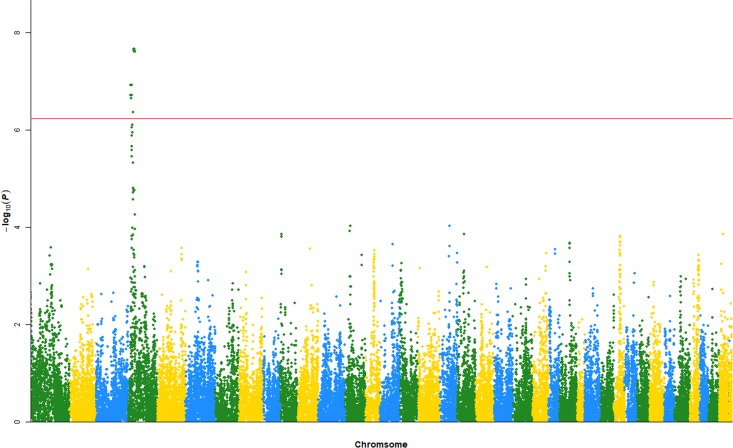
Manhattan plot of the replicated GWAS experiment

By using the NCBI SNP database (ftp://ftp.ncbi.nih.gov/snp/organisms/dog_9615/chr_rpts/), 44,542 SNPs were mapped to dog genes. Among them, the top 82 SNPs passed the Bonferroni thresholded *P*-value of 1.12e-6, and the top 45 SNPs passed the significance test with the empirical thresholded *P*-value of 5e-8. These results are shown in Figure 1 and Table 1, and the full dataset is shown in Supplementary Table 1.

**Table 1:** Results that reached genome-wide significance in Hayward et al.’s study [27] and our replicated GWAS experiment

Disease	Case/Control	Name of breeds	Hayward et al. [27] Top markers (chr:position)	Our replication Top markers (chr:position)
Idiopathic epilepsy	34/168	Irish Wolfhound	4:7.5–21	4:7.53–20.824

### Identification of significant subnetworks by HotNet2 and subsequent gene ontology analysis

Using automated procedures, HotNet2 identified 26 significant subnetworks (*P* = 0.02, Supplementary Table 2), which contained a total of 370 genes. A GO enrichment analysis of the genes was performed for each subnetwork (http://www.geneontology.org). Intriguingly, the results of the GO analysis from 4 of the 26 subnetworks were associated with epilepsy, as shown in Table 2.

**Table 2:** Results of the subnetwork GO analyses

Subnetwork index	Gene ontology analysis	Genes	*P*-value
1	GO molecular function complete		
	protein tyrosine phosphatase activity	PTPRD, PTPRK, PTPRM, PTPRT, EYA3	0.0126
4	GO biological process complete		
	amino acid transmembrane transport	SLC36A3, SLC1A2, SLC38A2, SLC7A8, SLC38A10, SLC38A11	6.06E-06
11	GO molecular function complete		
	cAMP response element binding	CREB3L1, CREB3L2	0.0316
17	GO cellular component complete		
	sarcoglycan complex	SGCD, SGCZ, SGCE	1.77E-06
	uniplex complex	MCU, MICU1	0.00202

The first subnetwork was the largest, containing 50 genes. The enrichment term used was “protein tyrosine phosphatase activity,” which is a significant molecular function, as previous studies have shown that mutations in protein tyrosine phosphatase can cause progressive myoclonus epilepsy [37–39]. Specifically, the genes involved in protein tyrosine phosphatase activity in this subnetwork are *PTPRD*, *PTPRK*, *PTPRM*, *PTPRT* and *EYA3*. Among these five genes, *PTPRM* is associated with mesial temporal lobe epilepsy [30], and *PTPRD* is implicated in the neurological disorder restless legs syndrome (RLS) [32].

*PTPRM* was reported to be involved in mesial temporal lobe epilepsy [30]. *PTPRM* encodes a cell surface receptor that is a homophilic cell-cell adhesion molecule expressed in neuronal, glial and endothelial cells [40]. *PTPRM* is part of the network associated with cell growth and differentiation mechanisms [41] and is also associated with neurite outgrowth [42]. Santos *et al* quantified *PTPRM* transcripts in hippocampal samples from patients and controls and observed up-regulated *PTPRM* transcript levels in the patient group compared with the control group [30]. Their results indicated involvement of *PTPRM* in the predisposition to mesial temporal lobe epilepsy.

Some evidence has shown that variants in the 5′ UTR of *PTPRD* are implicated in the neurological disorder RLS. These RLS-associated SNPs consist of ten noncoding exons contained in two known long splice variants expressed predominantly in fetal and adult brain tissue [32]. *PTPRD* encodes protein tyrosine phosphatase receptor type D, and it is associated with the regulation of synapse development and function [43]. RLS is the same neurological disease as epilepsy, and therefore, *PTPRD* may play a role in epilepsy.

**Figure 2:**
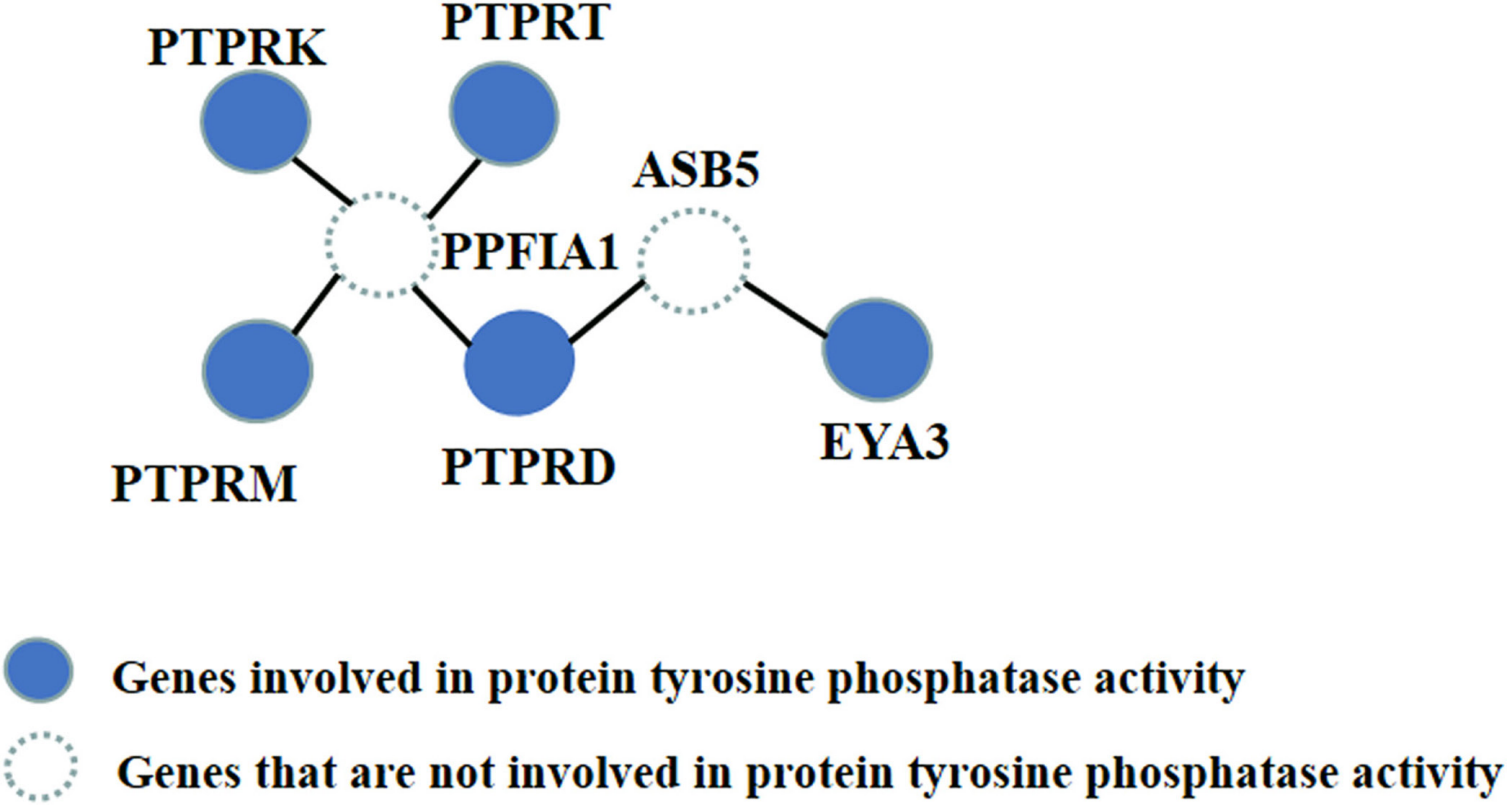
The topologies of genes involved in protein tyrosine phosphatase activity

Although the *P*-values of *PTPRM* and *PTPRD* were 0.0816 and 0.0723, respectively, which were not significant in the GWAS, they were highlighted by HotNet2. The other three protein tyrosine phosphatases were not associated with any relevant reports but may be involved in epilepsy etiology and deserve further study. Along with the other four genes, *PTPRD* was associated with two other latent vital genes discovered by HotNet2, *PPFIA1* and *ASB5*, which are marked as two dashed circles in Figure 2. *PPFIA1* encodes the Liprin-a1 protein, which contains 1202 amino acid residues with a molecular weight of approximately 136 kDa. Yin *et al* first reported that Liprin-a1 protein exists in the temporal neocortex of intractable epilepsy patients. They found that the expression level of Liprin-a1 was significantly higher in intractable temporal lobe epilepsy patients than in controls. Their results suggested that increased expression of Liprin-a1 in the brain is associated with human intractable epilepsy [28]. In addition, *ASB5* encodes a protein in the ankyrin repeat and SOCS box-containing (ASB) family of proteins. Novel copy number variations (CNVs) in *ASB5* have been identified in children with autism [33]. Davis *et al* found a 166-kb deletion on chromosome 4q34.2 in two of three trizygotic triplets, and this deletion included *ASB5*. Another study reported that deletions in this chromosome region are associated with several features including mild mental retardation, velo-cardio-facial (VCF) syndrome-like features, and finger clinodactyly [33]. Considering that autism and epilepsy are both neurological diseases, these results demonstrate the effectiveness of this method.

The fourth subnetwork contained 31 genes. The GO biological process results are shown in Table 2. The amino acid transmembrane transport function gene results yielded *SLC36A3*, *SLC1A2*, *SLC38A2*, *SLC7A8*, *SLC38A10*, and *SLC38A11*. All these genes transcribe proteins in the solute carrier family. Mutations in *SLC1A2* are critical causes of epileptic encephalopathies [29]. *SLC1A2* encodes one of the major glutamate transporters; mutations in *SLC1A2* in mice cause impaired glutamate uptake, and the resulting excess glutamate leads to subsequent excitotoxicity. The previous study defined a type of SLC1A2 encephalopathy and highlighted the importance of *SLC1A2* as a glutamatergic gene associated with epileptic encephalopathies [29]. The other genes, *SLC36A3*, *SLC38A2*, and *SLC7A8*, are associated with the transport of anti-epilepsy drugs [44–46].

HotNet2 also identified a subnetwork containing eleven proteins. The GO molecular functions are presented in Table 2. Studies have shown that inhibition of cAMP response element binding protein (CREB) transcription can shorten the duration of status epilepticus [47]. In this subnetwork, CREB3L1 and CREB3L2 were classified as belonging to the CREB protein family, and therefore, their mutations may regulate CRE transcription and appear to play a role in epilepsy.

The seventeenth subnetwork included eight genes, and the GO cellular components are listed in Table 2. The three genes involved in the sarcoglycan complex were *SGCD*, *SGCZ*, and *SGCE*. Interestingly, though SGCE was not significantly associated with epilepsy based on the GWAS method alone (*P* = 0.0517), it was highlighted in our method. In fact, it has been reported that epsilon-sarcoglycan (SGCE) mutations can lead to seizures in myoclonus-dystonia patients [31]. This gene encodes the epsilon member of the sarcoglycan family. Sarcoglycans are transmembrane proteins that are components of the dystrophin-glycoprotein complex, which link the actin cytoskeleton to the extracellular matrix. Studies have indicated that loss-of-function mutations in the SGCE gene are found in approximately 50% (range 20–80%) of epsilon-sarcoglycan cases [34, 48]. The mitochondrial calcium uniporter (MCU), which is associated with the uniplex complex, is also involved in many neurological diseases [49]. There is evidence demonstrating that mitochondrial dysfunction contributes to the pathophysiology of epilepsy [50, 51]. The MCU localizes to the inner membrane and is widely accepted to be responsible for calcium uptake by mitochondria [52]. Wang *et al* investigated the role of MCU in the rat pilocarpine model of epilepsy, which replicates the key features of temporal lobe epilepsy, and they found that MCU inhibition exerted a neuroprotective effect on seizure-induced brain injury through the mitochondria/ROS/CytC pathway [49].

In summary, the systems genetics analysis performed here broadened our understanding of mutations in epilepsy. Some gene mutations in these subnetworks have been reported to be significant in epilepsy. These results illustrate the effectiveness of the systems genetics method in determining the pathogenesis of epilepsy.

### Drug enrichment analysis of HotNet2 subnetworks

To test the effectiveness of the HotNet2 method in drug discovery, we counted the enrichment of the drugs in these subnetworks. We collected and sorted 5,452 active drugs corresponding to 2,440 targets in three databases: DrugBank (https://www.drugbank.ca), Therapeutic Target Database, (TTD, http://bidd.nus.edu.sg/group/cjttd/), and ClinicalTrials (https://www.clinicaltrials.gov). Among them, there were 79 drugs for the treatment of epilepsy, generating 226 targets. The HotNet2 results based on the relationships of drugs and their targets included 370 genes. We were able to identify 221 active drugs, of which 22 are used for the treatment of epilepsy (Table 3). The results of a hypergeometric test were significant (*P* = 2.130943e-13), indicating that the HotNet2 approach can markedly enrich anti-epilepsy drugs.

**Table 3:** Drug enrichment of subnetworks

Subnetwork index	Drugs	Targets
2	Adinazolam	GABRP
	Clobazam	GABRP
	Clonazepam	GABRP
	Diazepam	GABRP
	Lorazepam	GABRP
	Meprobamate	GABRP
	Metharbital	GABRP
	Nitrazepam	GABRP
	Primidone	GABRP
	Propofol	GABRP
	Topiramate	GABRP
	Lacosamide	SCN10A
	Valproic Acid	SCN10A
4	Felbamate	GRIN3A
	Gabapentin	GRIN3A
	Ketamine	GRIN3A
	Phenobarbital	GRIN3A
14	CPP-15	ABAT
	DP-VPA	ABAT
	Tiagabine	ABAT
	Valproic acid	ABAT
	Vigabatrin	ABAT
19	Zonisamide	CA13

We also examined the drug enrichment of a single genetic locus in the GWAS results. We selected the most significant *P*-value of the first 370 genes and obtained 248 active agents, including 15 epilepsy agents. A *P*-value of 1.912487e-06 was obtained using the hypergeometric test. By comparison, the capacity of the HotNet2 method to enrich anti-epilepsy drugs was dramatically higher than that of the GWAS method.

### Potential combinatorial anti-epilepsy drugs

If drugs that treat epilepsy are enriched in the same subnetwork and correspond to different targets, these drugs may be more effective in combination. Through drug enrichment, the second subnetwork contained 13 drugs, of which 11 drugs corresponded to the target GABRP, and the other two drugs corresponded to the target SCN10A. Therefore, we considered 22 potential combinations of anti-epilepsy drugs (Table 4). In PubMed, we counted the number of co-occurrences of these combinations of drugs and the keyword “epilepsy” in abstracts. As shown in Table 4, six of the drug combinations were found in a total of more than 100 abstracts. In addition, in the Drug Combination database (DCDB, https://omictools.com/drug-combination-database-tool), we found that the combination of topiramate and valproic acid (Drug Combination ID: DC000445) has been reported to play a role in anti-epileptic treatment, indicating that this method for identifying drug combinations is reliable.

**Table 4:** Potential drug combinations for epilepsy

Index	Potential combinations of drugs	Count
1	Adinazolam + Lacosamide	0
2	Clobazam + Lacosamide	17
3	Clonazepam +Lacosamide	10
4	Diazepam + Lacosamide	13
5	Lorazepam + Lacosamide	12
6	Meprobamate + Lacosamide	0
7	Metharbital + Lacosamide	0
8	Nitrazepam + Lacosamide	2
9	Primidone + Lacosamide	7
10	Propofol + Lacosamide	9
11	Topiramate + Lacosamide	58
12	Adinazolam + Valproic Acid	0
13	Clobazam + Valproic Acid	142
14	Clonazepam + Valproic Acid	457
15	Diazepam + Valproic Acid	407
16	Lorazepam + Valproic Acid	115
17	Meprobamate + Valproic Acid	9
18	Metharbital + Valproic Acid	1
19	Nitrazepam + Valproic Acid	35
20	Primidone + Valproic Acid	272
21	Propofol + Valproic Acid	41
22	Topiramate + Valproic Acid	521

## MATERIALS AND METHODS

### Data & materials

The data were from a study by Hayward *et al.* [27], an extensive dog genotyping dataset with 4,224 samples genotyped on a semicustom 180,000 SNP array for several complex traits. Among the most common recorded diseases collected in these data, there were 7 across-breed phenotypes (canine hip dysplasia, elbow dysplasia, cranial cruciate ligament disease, mast cell tumor, lymphoma, portosystemic vascular anomalies and mitral valve degeneration) and 5 within-breed phenotypes (idiopathic epilepsy in Irish Wolfhounds, granulomatous colitis in Boxers and Bulldogs, lymphoma in Golden Retrievers, mast cell tumor in Labrador Retrievers and portosystemic vascular anomalies in Yorkshire Terriers). To avoid the diversity between across-breed individuals, we retained the disease idiopathic epilepsy (IE) in Irish Wolfhounds, which consisted of 200 individuals (34 cases and 168 controls) and 160,727 SNPs. Case dogs were diagnosed by metabolic screening, magnetic resonance imaging and EEG, while control dogs were older than 5 years and without a history of seizures.

The raw GWAS data were preprocessed by PLINK v1.9 (http://zzz.bwh.harvard.edu/plink/tutorial.shtml). Only SNPs with a minor allele frequency (MAF) > 0.05 and Hardy–Weinberg test (HWE) < 0.001 in these individuals were included. After quality control, 89,085 SNPs remained.

### Replication of the GWAS experiment using multiple logistic regression

Logistic regression (LR) is a suitable algorithm for associating phenotypes with different genotypes. Assuming *Y* is the binary genotype variable, and *x* is the phenotype variable (continuous or discrete), we can obtain the association of *y* and *x* by LR. The goal of LR is to model p(x)≡P(Y=1|x). In LR, the probability p(x) is modeled by $p\left(x\right)=\frac{\exp\left(\alpha +\beta x\right)}{1+\exp\left(\alpha +\beta x\right)}$, which can be conveniently converted to $\log\left(\frac{p\left(x\right)}{1-p\left(x\right)}\right)=\alpha +\beta x$. To test $H_{0}:\beta =0$ vs. $H_{1}:\beta \neq 0$, the test statistic $Z=\frac{\hat{\beta }}{\sqrt{\hat{V}a\left(\hat{\beta }\right)}}$ is used.

To match the multinomial nature of the genotype data, we use multinomial logistic regression (MLogit, or multiclass logistic regression [36]) to calculate the association of *y* and *x*. We denote the genotype variable *y* = 0, 1, and 2, and the MLogit model is as follows:

$$Q_{1}=P\left(y=0|x\right)=\frac{\exp\left(\alpha_{1}+\beta x\right)}{1+\exp\left(\alpha_{1}+\beta x\right)}$$

$$Q_{2}=P\left(y\leq 1|\vec{x}\right)=\frac{\exp\left(\alpha_{2}+\beta x\right)}{1+\exp\left(\alpha_{2}+\beta x\right)}$$

$$Q_{3}=P\left(y\leq 2|x\right)=1-Q_{2}$$

$$p_{1}=P\left(y=0|x\right)=Q_{1}$$

$$p_{2}=P\left(y=1|x\right)=Q_{2}-Q_{1}$$

$$p_{3}=P\left(y=2|x\right)=Q_{3}-Q_{2}$$

We can estimate the parameters $\alpha ,\beta_{1},\beta_{2}$ using the maximum likelihood method and obtain the probabilities *p*_*1*_, *p*_*2*_ and *p*_*3*_. In this study, we used the R package “MLogit” [36] to obtain the estimators.

### Using the HotNet2 algorithm to find subnetworks related to epilepsy

HotNet2 (HotNet diffusion-oriented subnetworks) performs well for finding significant subnetworks, which consist of genes with a higher mutation tendency than expected by chance in a large gene interaction network. HotNet2 is based on a heat diffusion kernel algorithm by considering random walks with restarts [53].

The main inputs to HotNet2 include a heat vector, which contains the score of each gene and the interactions among the corresponding gene pairs. First, to compute the heat vector for each gene, the sorted *P*-values of SNPs in the GWAS were used after removing redundancy. Specifically, the minimum GWAS *P*-value of the SNPs in the gene was set as the final heat value of the gene. Thus, 12,355 genes with their corresponding *P*-values were obtained. To avoid information loss of the amount of gene mutations, Spearman testing was performed to indicate the positive correlations between the number of gene mutations and the negative logarithm of the minimum *P*-value (i.e., credibility). Here, the correlation value for Spearman testing was 0.294, and the credibility was 9.0459e-245, i.e., the minimum *P*-value contains information on the number of mutations. Afterwards, the top 20% of genes (*n* = 2471) were selected as the input, and the negative log base 10 of each *P*-value was used for the computation formula. Second, to obtain the interactions used for HotNet2, *canis lupus familiaris* protein-protein interaction networks were derived from the STRING database (STRING, http://www.string-db.org). As the default score threshold is usually 400 [54], interactions with scores lower than 400 were discarded.

The parameter in HotNet2 was set as the default, β = 0.4, as cited by Leiserson *et al* [53]. Finally, the most significant (*P* < 0.05) subnetworks were identified.

### Study flowchart

**Figure 3:**
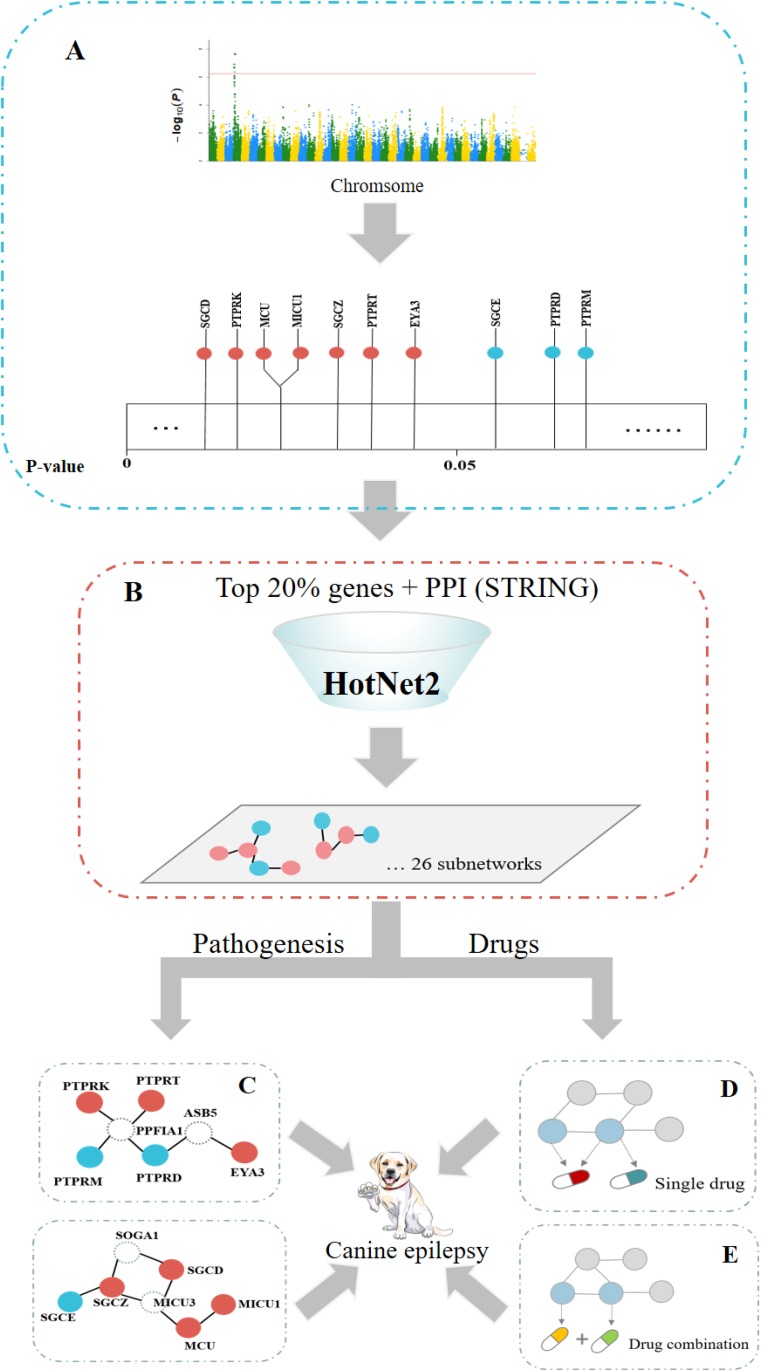
Study flowchart (**A**) The associations of genes with canine epilepsy were calculated by the GWAS method. Some genes were significantly associated with canine epilepsy (*P* < 0.05, marked with orange), and some were not (marked by blue in the figure). (**B**) The significant subnetworks related to canine epilepsy were found using the HotNet2 method. (**C**) GO enrichment analysis of each subnetwork led to enhancement of the GWAS results and allowed exploration of the pathogenesis of canine epilepsy. (**D**) Relationship statistics of single drug-gene pairs that passed the enrichment testing of the anti-epilepsy drugs in these subnetworks. (**E**) These drug combinations are likely to produce synergistic effects if separate target genes of different drugs co-occur in a vital HotNet2 subnetwork.

A flowchart of this study is shown in Figure 3.

## CONCLUSIONS

The domestic dog (*Canis familiaris*) is not only a faithful partner of humans but also an excellent model animal for studying human diseases, as the dog genome and clinical manifestations are extremely similar to those of humans [7]. In this study, the genotype and epilepsy phenotype data of Hayward *et al.* [27] were used to explore the pathogenesis of canine epilepsy in a combined method using GWAS and HotNet2.

Using the combination of GWAS and HotNet2, we ultimately identified 26 subnetworks associated with canine epilepsy. Through GO enrichment analysis and a literature review, we focused on the relationship between gene mutations and epilepsy mechanisms in four subnetworks. We automatically identified *PTPRD*, *PPFIA1*, *ASB5*, *SLC1A2*, and *SGCE* from a large-scale interaction network. *PTPRM*, *PTPRD* and *SGCE* were of particular interest, as they were not significant results of the GWAS and, thus, demonstrated the power of this method. Moreover, *PTPRM*, *PPFIA1*, *SLC1A2*, and *SGCE* were confirmed to be associated with epilepsy, while *PTPRD*, *ASB5*, and *MCU* were involved in neurological disorders. This evidence suggests that the method for key gene discovery is reliable.

In addition, we performed drug enrichment on each subnetwork, resulting in 221 active drugs, of which 22 were drugs used for the treatment of epilepsy. The hypergeometric test was extremely significant and was stronger than the single-site GWAS method of drug enrichment. If targets of agents correspond to different target genes in the same subnetwork, their combinations are likely to induce synergistic effects. We found 22 potential combinations of anti-epilepsy drug treatments. One of these drug combinations, topiramate and valproic acid (DrugCombination_ID: DC000445), have been confirmed by the DCDB database for the treatment of epilepsy.

In summary, this study demonstrated that the combined GWAS and HotNet2 methods can effectively exploit the pathogenic factors of epilepsy and be used to identify potential drug combinations. Moreover, not only is the present approach helpful for epilepsy, but it will also be useful for exploring the pathogenic mechanisms of other diseases and potential drug combinations.

## SUPPLEMENTARY MATERIALS TABLES






