# Supplementary material for: Exploring the pathogenesis of canine epilepsy using a systems genetics method and implications for anti-epilepsy drug discovery
Source: Oncotarget. 2017 Dec 27;9(17):13181–92. doi: 10.18632/oncotarget.23719 (PMC5862570; doi:10.18632/oncotarget.23719)
Supplement: Supplementary file 1 [file oncotarget-09-13181-s001.pdf]

Exploring the pathogenesis of canine epilepsy using a systems genetics method and implications for anti-epilepsy drug discovery

SUPPLEMENTARY MATERIALS

Supplementary Table 1: GWAS Replicate experiments and mapped dog genes. See_Supplementary_Table 1

Supplementary Table 2: 26 significant subnetworks associated with canine epilepsy. See_Supplementary_Table 2
